# Efficacy of loading dose colistin versus carbapenems for treatment of extended spectrum beta lactamase producing *Enterobacteriaceae*

**DOI:** 10.1038/s41598-020-78098-4

**Published:** 2021-01-08

**Authors:** Wasan Katip, Jukapun Yoodee, Suriyon Uitrakul, Peninnah Oberdorfer

**Affiliations:** 1 Department of Pharmaceutical Care, Faculty of Pharmacy, Chiang Mai University, Chiang Mai, 50200 Thailand; 2 Epidemiology Research Group of Infectious Disease (ERGID), Chiang Mai University, Chiang Mai, 50200 Thailand; 3 Department of Pharmaceutical Care, School of Pharmacy, Walailak University, Nakhon Si Thammarat, 80160 Thailand; 4 Division of Infectious Diseases, Department of Pediatrics, Faculty of Medicine, Chiang Mai University, Chiang Mai, 50200 Thailand

**Keywords:** Diseases, Medical research

## Abstract

Colistin provides in vitro activity against numerous ESBL-producing and carbapenem-resistant bacteria. However, clinical information with respect to its utilization in infection caused by ESBL producers is limited. The aim of this study was a comparison of mortality rates of loading dose (LD) colistin and carbapenems as definitive therapies in a cohort of patients with infections caused by ESBL-producing *Escherichia coli* and *Klebsiella pneumoniae*. A retrospective cohort study in 396 patients with ESBL-producing *E.coli* and *K.pneumoniae* infection at a university-affiliated hospital was conducted between 1 January 2005 and 30 June 2015 to compare outcomes of infected patients who received LD colistin (95 patients) with carbapenems (301 patients). The three primary outcomes were 30-day mortality, clinical response and microbiological response. The most common infection types were urinary tract infection (49.49%), followed by pneumonia (40.66%), bacteremia (13.64%), skin and soft tissue infections (4.80%) and intra-abdominal infection (3.03%). LD colistin group provided higher 30-day mortality when compared with carbapenems group (HR 7.97; 95% CI 3.68 to 17.25; *P* = 0.001). LD colistin was also independently associated with clinical failure (HR 4.30; 95% CI 1.93 to 9.57; *P* = 0.001) and bacteriological failure (HR 9.49; 95% CI 3.76 to 23.96; *P* = 0.001) when compared with those who received carbapenems. LD colistin treatment was associated with poorer outcomes, i.e. mortality rate, clinical response and microbiological response. Moreover, when adjusted confounding factors, LD colistin was still less effective than carbapenems. It should be noted that, however, the use of Vitek-2 to assess colistin susceptibility could provide inaccurate results. Also, the difference in baseline characteristics could still remain in retrospective study although compensation by hazard ratio adjustment was performed. Therefore, clinical utilization of LD colistin should be recommended as an alternative for treatment ESBL-producing *Enterobacteriaceae* only in the circumstances where carbapenems cannot be utilized, but this recommendation must be considered carefully.

## Introduction

Extended-spectrum β-lactamase (ESBL)-producing *Enterobacteriaceae* has emerged worldwide and has become important nosocomial infection. A retrospective cohort study that was conducted in Sa Kaeo and Nakhon Phanom provinces, Thailand, reported that the overall mortality rate of bloodstream infections due to ESBL-producing *Enterobacteriaceae* from 2008 and 2014 was 20.0%^1^.

ESBL production limits the number of drugs that can be used for an effective treatment of an infection^2^. However, the effectiveness of antimicrobial agents for the treatment of ESBL-producing *Enterobacteriaceae* is controversial^2,3^. In a multinational study of 85 patients with ESBL-producing *Klebsiella pneumoniae* bacteremia, carbapenems provided the lowest 14-day mortality^4^. Moreover, a meta-analysis from non-randomized studies compared mortality rates between carbapenems and alternative antibiotics for the treatment of ESBL-positive *Enterobacteriaceae* bacteraemia, and reported lower mortality when using carbapenems than alternative antibiotics for definitive and empirical treatment^5^. Therefore, carbapenems have become the standard treatment of ESBL-producing *Enterobacteriaceae*^2,3,5^.

Colistin or polymyxin E is an old antimicrobial agent which was discovered from *Bacillus polymyxa*, subspecies Colistinus Koyama. In 1949, this agent was used in the form of colistimethate sodium to treat infections caused by extensively drug-resistant Gram-negative bacteria. (XDR-GNB)^6,7^. Colistin is one of antibiotics that exhibited excellent in vitro efficacy against ESBL-producing *Enterobacteriaceae*^8^. There has been a few case reports of successful use of polymyxins to treat ESBL-associated infections, and to date only a few clinical studies concerning the efficacy of colistin for treatment of infections with ESBL-producing *Escherichia coli* and *K. pneumoniae*^9,10^. Therefore, the aim of this study was to evaluate clinical efficacy of loading dose (LD) colistin compared with carbapenems for treatment ESBL-producing *Escherichia coli* and *Klebsiella pneumoniae*.

## Results

A total of 396 cases were included; 95 patients received with LD colistin and 301 patients with carbapenems (241 patients with meropenem, 40 with ertapenem and 20 with imipenem). Characteristics of patient infection caused by ESBL-producing *Enterobacteriaceae* are shown in Table 1. The minimum inhibitory concentrations (MICs) of colistin against all 95 isolates of *K. pneumoniae* and *E. coli* strains in the LD colistin group were lower than 0.5 µg/mL.

**Table 1:** Characteristics of patient infection caused by ESBL-producing *E. coli* and *K. pneumoniae.*

Characteristic	Definitive therapy cohort		
	LD colistin (n = 95)	Carbapenems (n = 301)	*p* value
Age (yr) (mean ± SD)	65.04 ± 16.60	62.96 ± 19.82	0.357
Female	61 (64.21)	148 (49.17)	0.013^a^
ICU admission	64 (67.37)	44 (14.62)	0.001^a^
Mechanical ventilation	75 (78.95)	251 (83.39)	0.355
Duration of hospitalization, median (min–max)	34 (4–157)	12 (5–58)	0.001^a^
Appropriate antibiotic treatment	84 (88.42)	295 (98.01)	0.001^a^
Charlson score (mean ± SD)	2.73 ± 2.35	4.43 ± 2.22	0.001^a^
**Underlying diseases**			
Cerebrovascular disease	29 (30.85)	175 (58.14)	0.001^a^
Diabetes mellitus	20 (21.05)	92 (30.56)	0.089^a^
Chronic kidney disease	21 (22.34)	60 (20.00)	0.661
Malignancy	25 (26.60)	93 (30.90)	0.519
COPD	10 (10.53)	38 (12.62)	0.719
Liver disease	9 (9.57)	17 (5.65)	0.231
**Infection type**			
Pneumonia	45 (47.37)	116 (38.54)	0.150^a^
Urinary tract infection	31 (32.63)	165 (54.82)	0.001^a^
Bloodstream infection	41 (43.16)	13 (4.32)	0.001^a^
Skin and soft-tissues, surgical sites, joints, and bones	13 (13.68)	6 (1.99)	0.001^a^
Intra-abdominal infection	5 (5.26)	7 (2.33)	0.169^a^
Duration of treatment (mean ± SD)	8.47 ± 4.86	11.17 ± 3.95	0.001^a^
**Pathogen causing infection**			
*K. pneumoniae* ESBL	45 (47.37)	147 (48.84)	0.815
*E.coli* ESBL	55 (57.89)	154 (51.16)	0.289
Nephrotoxicity	46 (48.42)	0 (0.00)	0.001^a^

^a^Variable used to adjustment for treatment with LD colistin.

The overall 30-day mortality was significantly higher in patients receiving definitive therapy with LD colistin compared to carbapenems (51.58% and 25.42%, respectively, *P* = 0.001). A multivariate model indicated significant association between LD colistin and mortality as compared with carbapenems (HR 7.97; 95% CI 3.68 to 17.25; *P* = 0.001) after adjustment for gender, ICU admission, duration of hospitalization, appropriate antibiotic treatment, Charlson comorbidity score, cerebrovascular disease, diabetes mellitus, infection type and nephrotoxicity. LD colistin was also independently associated with clinical failure (HR 4.30; 95% CI 1.93 to 9.57; *P* = 0.001) and bacteriologic failure (HR 9.49; 95% CI 3.76 to 23.96; *P* = 0.001) when adjusted for gender, ICU admission, use mechanical ventilation, duration of hospitalization, appropriate antibiotic treatment, Charlson comorbidity score, cerebrovascular disease, diabetes mellitus, infection type (i.e. pneumonia, urinary tract infection, bloodstream infection, skin and soft-tissue infections, infection at surgical sites, infection at joints and bones, and intra-abdominal infection) and nephrotoxicity (Table 2).

**Table 2:** Cox regression analysis of outcomes of patients treated with LD colistin and carbapenems for the infection caused by ESBL-producing *E. coli* and *K. pneumoniae.*

Outcome parameter	No. of patients (%) with each outcome with indicated treatment		Unadjusted HR (95% CI)	*p* value	Adjusted HR* (95% CI)	*p* value
	LD colistin (n = 95)	carbapenems (n = 301)				
30-day mortality	49 (51.58)	76 (25.42)	2.94 (2.05–4.22)	0.001	7.97 (3.68–17.25)	0.001
Clinical failure	38 (40.00)	83 (27.57)	2.14 (1.45–3.14)	0.001	4.30 (1.93–9.57)	0.001
Bacteriological failure	30 (31.58)	64 (21.26)	2.15 (1.39–3.33)	0.001	9.49 (3.76–23.96)	0.001

* Adjusted for gender, ICU admission, use mechanical ventilation, duration of hospitalization, appropriate antibiotic treatment, Charlson comorbidity score, cerebrovascular disease, diabetes mellitus, infection type and nephrotoxicity.

CI, confidence interval; HR hazard ratio.

The overall 30-day mortality rates were still higher in patients receiving definitive therapy with LD colistin compared to carbapenems, after subgroup analysis with type of carbapenems, i.e. meropenem, ertapenem and imipenem (Table 3), and with the infection type (Table 4).

**Table 3:** Cox regression analysis of 30-day mortality of patients treated with LD colistin and carbapenems (meropenem, ertapenem, imipenem) for the infection caused by ESBL-producing *E. coli* and *K. pneumoniae.*

Variable	No. of patients (%) with 30-day mortality	Unadjusted HR (95% CI)	*p* value	Adjusted HR* (95% CI)	*p* value
Meropenem (n = 241)	60 (24.90)	1.00 (Reference)		1.00 (Reference)	
LD colistin (n = 96)	49 (51.58)	3.05 (2.09–4.45)	0.001	6.28 (2.96—13.29)	0.001
Ertapenem (n = 40)	12 (30.00)	1.00 (Reference)		1.00 (Reference)	
LD colistin (n = 96)	49 (51.58)	2.43 (1.23–4.82)	0.011	7.58 (2.85–20.14)	0.001
Imipenem (n = 20)	6 (30.00)	1.00 (Reference)		1.00 (Reference)	
LD colistin (n = 96)	49 (51.58)	2.77 (1.10–6.99)	0.030	7.03 (2.15–23.03)	0.001

* Adjusted for gender, ICU admission, use mechanical ventilation, duration of hospitalization, appropriate antibiotic treatment, Charlson comorbidity score, cerebrovascular disease, diabetes mellitus, infection type and nephrotoxicity.

CI, confidence interval; HR hazard ratio.

**Table 4:** Cox regression analysis of 30-day mortality of patients treated with LD colistin and carbapenems for the infection types caused by ESBL-producing *E. coli* and *K. pneumoniae.*

Infection types	No. of patients (%) with 30-day mortality	Unadjusted HR (95% CI)	*p* value	Adjusted HR* (95% CI)	*p* value
**Pneumonia**					
Carbapenems (n = 116)	46 (39.66)	1.00 (Reference)		1.00 (Reference)	
LD colistin (n = 45)	22 (48.89)	1.52 (0.91–2.54)	0.107	8.38 (2.73–25.72)	0.001
**Urinary tract infection**					
Carbapenems (n = 165)	31 (18.79)	1.00 (Reference)		1.00 (Reference)	
LD colistin (n = 31)	17 (54.84)	4.43 (2.43–8.07)	0.001	6.64 (1.80–24.58)	0.005
**Bloodstream infection**					
Carbapenems (n = 13)	4 (30.77)	1.00 (Reference)		1.00 (Reference)	
LD colistin (n = 41)	23 (56.10)	3.17 (1.07–9.40)	0.038	9.51 (1.48–60.97)	0.018

* Adjusted for gender, ICU admission, use mechanical ventilation, duration of hospitalization, appropriate antibiotic treatment, Charlson comorbidity score, cerebrovascular disease, diabetes mellitus, infection type and nephrotoxicity.

CI, confidence interval; HR hazard ratio.

**Figure 1:**
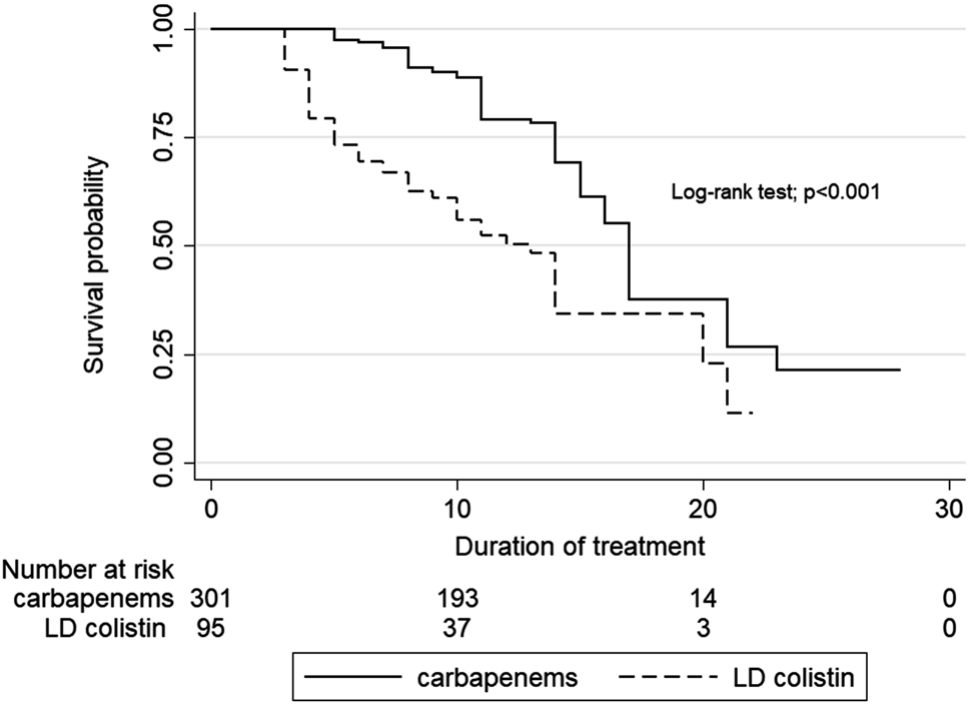
Kaplan–Meier curve showing survival probability of infection caused by ESBL-producing *E. coli* and *K. pneumoniae* according to LD colistin and carbapenems regimens.

Univariate cox-regression analysis showed that definitive therapy with LD colistin and ICU status were associated with increased mortality. In the multivariable analysis carried out with Cox regression, only definitive therapy with LD colistin showed association with mortality. However, underlying disease was also independently associated with clinical failure (Table 5). Mortality rates were significantly higher in patients receiving definitive therapy with LD colistin as compared to carbapenems (*P* < 0.001 by log-rank test) (Fig. 1).

**Table 5:** Cox regression analysis of associations between different variables and primary outcomes including 30-day mortality, clinical response and bacteriological response.

Variable	Univariable analysis			Multivariable analysis		
	Unadjusted HR	HR (95%CI)	p-value	Adjust HR	HR (95%CI)	p-value
**30-day mortality**						
Definitive therapy with LD colistin	2.94	2.05–4.22	0.001	3.14	1.75–5.62	0.001
ICU status	1.91	1.33–2.73	0.001	1.05	0.66–1.66	0.838
Charlson score ≥ 4	0.91	0.64–1.30	0.621	1.13	0.77–1.65	0.546
Age ≥ 60	1.47	0.99–2.18	0.054	1.49	0.99–2.23	0.054
Underlying disease	1.49	0.80–2.78	0.203	1.51	0.80–2.85	0.206
Nephrotoxicity	2.48	1.60–3.82	0.001	0.10	0.56–1.77	0.996
**Clinical failure**						
Definitive therapy with LD colistin	2.14	1.45–3.15	0.001	2.20	1.16–4.16	0.016
ICU status	1.28	0.87–1.88	0.205	0.82	0.50–1.33	0.432
Charlson score ≥ 4	0.75	0.52–1.07	0.117	0.83	0.56–1.21	0.336
Age ≥ 60	1.09	0.75–1.59	0.641	1.02	0.69–1.50	0.905
Underlying disease	2.13	1.04–4.36	0.039	2.35	1.13–4.91	0.022
Nephrotoxicity	2.20	1.38–3.49	0.001	1.16	0.61–2.22	0.654
**Bacteriological failure**						
Definitive therapy with LD colistin	2.16	1.39–3.33	0.001	2.76	1.38–5.50	0.004
ICU status	1.30	0.84–2.00	0.233	0.80	0.46–1.40	0.445
Charlson score ≥ 4	0.83	0.55–1.25	0.378	0.93	0.61–1.44	0.760
Age ≥ 60	1.04	0.68–1.60	0.842	0.97	0.63–1.50	0.885
Underlying disease	1.88	0.87–4.07	0.107	2.07	0.95–4.63	0.066
Nephrotoxicity	1.82	1.05–3.18	0.033	0.84	0.41–1.72	0.626

CI, confidence interval; HR hazard ratio.

## Discussion

The clinical studies of colistin for the treatment of ESBL-producing *Escherichia coli* and *Klebsiella pneumoniae* are limited. This study was to evaluate clinical efficacy of loading dose (LD) colistin compared with carbapenems for treatment ESBL-producing *Escherichia coli* and *Klebsiella pneumoniae*. The unadjusted 30-day mortality was higher among LD colistin-treated patients than carbapenems-treated patients. The adjusted analysis showed higher mortality in the LD colistin group that was statistically significant in patients with ESBL-producing *E. coli* and *K. pneumoniae*. LD colistin was also independently associated with clinical failure and bacteriological failure. Based on these results, colistin was less effective than carbapenems. Thus, colistin should be reserved only for treatment of infections that are resistant to all antibiotics including carbapenems.

There are no strict guidelines or policies pertaining to the preferred therapeutic management of infections due to ESBL-producing *Enterobacteriaceae* although many health professionals have considered that carbapenems are the preferred agents^11^. Harris, et al. conducted a non-inferiority randomized clinical trial that included 391 patients with ESBL-producing *Enterobacteriaceae* infection. They reported that definitive treatment with piperacillin-tazobactam did not provide non-inferior 30-day mortality as compared to meropenem. These findings therefore did not support use of piperacillin-tazobactam in this condition^12^.

Colistin has promising *in-vitro* activity against ESBL-producing *Enterobacteriaceae* including *E. coli*, *K. pneumoniae* and *Enterobacter cloacae*^13^. Despite it has the in vitro activity, the use of colistin in ESBL-producing *Enterobacteriaceae* treatment is still debatable because of limited available evidences in human trials^13^.

Our study found that after controlling for confounding factors, LD colistin was associated with increased risk of death (HR 3.14; 95% CI 1.75 to 5.62; *P* < 0.001). Carbapenems were associated with significantly better clinical outcomes than the other antimicrobial, i.e. LD colistin. Moreover, the results from subgroup analysis with the infection type and type of carbapenems, found better survival rates of carbapenems in the subgroup analysis with infection types. This was similar to the findings from a study in patients with non-urinary ESBL bloodstream infections during 2010 and 2012. The multivariate analysis results showed that 10 patients who received piperacillin-tazobactam had higher 90-day mortality as compared to 69 patients who received a carbapenem (adjusted odds ratio, 7.9, *P* = 0.03)^14^. Likewise, a randomized study indicated that 100% (10/10) of the patients with nosocomial pneumonia who were treated with imipenem had clinical response while only 69% (9/13) of patients treated with cefepime had the same outcome^15^.

Our results might be explained by three reasons. Firstly, rapid emergence of resistance of colistin monotherapy has been reported in vitro using pharmacokinetic/pharmacodynamic models to mimic the pharmacokinetics of colistin in patients^16^. Moreover, several reports indicated that use of colistin for ESBL-producing *Klebsiella pneumoniae* infection could lead to an emergence of colistin resistance in *Klebsiella pneumoniae*^13,17^. There are several mechanisms dependent on the bacterial species, e.g. the mobile colistin resistance genes (*mcr*) in *E. coli* or the chromosomal mutations in regulatory genes of LPS synthesis in *K. pneumoniae*^18,19^. However, the emergence of colistin-resistant bacteria was not observed in the present study. Secondly, when given colistin systemically, this drug is unlikely to be effective for pneumonia because of its poor penetration into the pulmonary parenchyma^20^. In the present study, 47.37% of pneumonia patients were in the colistin group. Thirdly, the variation of LD colistin concentrations was high; it was found that patients who received the same loading doses of CBA (300 mg) had different colistin concentrations at a steady state^21^. Therefore, patients in this study might have different concentrations of colistin, resulting in different bacterial effects.

Nephrotoxicity is the main adverse effect reported with the use of colistin. In the present study, nephrotoxicity was found in 48.42% of the patients in LD colistin group but was not found in carbapenem group (*P* = 0.001). However, using multivariate Cox-regression analysis, nephrotoxicity was not associated with 30-day mortality (HR 0.10; 95% CI 0.56 to 1.77; *P* = 0.996), clinical failure (HR 1.16; 95% CI 0.61 to 2.22; *P* = 0.654), and bacteriological failure (HR 0.84; 95% CI 0.41 to 1.72; *P* = 0.626).

The present study has some limitations. Firstly, this study was the difference in baseline characteristics. This difference, however, was found in most retrospective studies. Although we attempted to adjust for potential confounders in the statistical methods, residual unknown confounding factors could remain with this study design. Furthermore, a complete compensation of the basic differences is probably not possible and the adjusted hazard ratios are subject to high uncertainty. So, the results should be interpreted with caution due to possible confounders and lack of some information.

Secondly, colistin should be reserved for specific situations which no other drugs can be used. Therefore, randomized clinical trial is not an appropriate study design for this purpose. However, this large retrospective study provides evidence regarding the efficacy of colistin in the treatment of ESBL-producing *Enterobacteriaceae*. So, until more definitive studies are performed, our findings suggested that LD colistin was inferior to carbapenem therapy for the treatment of ESBL-associated infections.

Thirdly, the appropriate methods for determination of colistin (polymyxin E) MIC have been discussed for several years. EUCAST and CLSI recently published a joint recommendation that broth microdilution (BMD) is the only validated method for antimicrobial susceptibility testing of colistin. Other testing methods such as agar dilution, disk diffusion and gradient diffusion are not currently recommended. However, the use of broth microdilution method for susceptibility testing may not be practical in routine diagnostic microbiology laboratories because of the individual laboratory workloads. Therefore, many laboratories still use alternative methods such as gradient strips and semi-automated systems in order to manage their workloads^22^.

Chiang Mai University Hospital had used Vitek-2 semi-automated system (bioMérieux) to test susceptibility since 2005 because Vitek-2 was, at the time, reported as an acceptable testing method for colistin^23–25^. Moreover, for *K. pneumoniae* and *E. coli*, the Vitek-2 provided better performance for isolates with MIC ≤ 0.5 and ≥ 16 µg/mL than the isolates with MIC within > 0.5 and < 16 µg/mL. These results were presented by Girardello et al.^26^ that compared performance of Vitek-2 with BMD in determination of colistin susceptibility. The authors^26^ reported a good correlation of results between Vitek-2 and BMD when tested *K. pneumoniae* and *E. coli* with isolates of MICs ≤ 0.5 and ≥ 16 µg/mL. In addition, the Vitek-2 was able to detect the resistance of 10 isolates of *mcr*-1-carrying *E. coli* although this polymyxin resistance mechanism exhibited borderline MICs of 4 µg/mL [6 isolates] and 8 µg/mL [4 isolates]. Therefore, Girardello et al. suggested that susceptibility testing with the reference method (broth microdilution) might be unnecessary when Vitek-2-determined MICs were either very low (≤ 0.5 µg/mL) or very high (≥ 16 µg/mL)^26^.

Consistently, Lo-Ten-Foe et al.^25^ compared the Vitek-2 colistin susceptibility test to the BMD reference test and showed a high level of agreement; there were only heteroresistant *E. cloacae* isolates which Vitek-2 failed to detect. Additionally, the study by Lellouche et al.^22^, which was performed in 274 isolates with colistin MIC ≤ 1 µg/mL by BMD, reported very good correlation between Vitek-2 testing and BMD. However, for the isolates with MIC > 1 µg/mL, Vitek-2 had poor correlation with the BMD as Vitek-2 yielded lower MICs.

With regards to the present study, the MICs of colistin against all 95 strains of ESBL-producing *K. pneumoniae* and *E. coli* in the LD colistin group were lower than 0.5 µg/mL. Therefore, the susceptibility of colistin in the present study was very likely to be acceptably reliable according to the above-mentioned results. Moreover, our study included only ESBL-producing *K. pneumoniae* and *E. coli,* but did not include carbapenem-resistant *Enterobacteriaceae* (CRE). It is known that MICs of ESBL-producing *K. pneumoniae* and *E. coli* were usually less than the MICs of CRE, so we were convinced that all isolates in our study were really sensitive to carbapenem.

Based on the information provided, ESBL infections in present study should not be misclassified as false-susceptible for colistin and treated with colistin.

## Conclusions

LD colistin treatment was associated with poorer survival rate compared to carbapenems. Adjusted analyses also suggested that LD colistin was less effective than carbapenems. Thus, patients who were infected with ESBL-producing *Enterobacteriaceae* should be firstly treated with carbapenems. LD colistin should be considered as an alternative to carbapenems for treatment of ESBL-producing *Enterobacteriaceae* only in the circumstances where carbapenems cannot be used such as infections with carbapenem-resistant Gram-negative pathogens, patients with history of type 1 hypersensitivity of penicillin, or allergic reaction to penicillin was severe (e.g. anaphylactic shock). However, the conclusion in this study was based on only retrospective data with differences in baseline characteristics. Although hazard ratio adjustment to compensate these differences was well performed, obviously it was not possible to completely remove all confounders. Therefore, interpreting the results from our study should be done very carefully.

## Methods

### Study setting and participants

A retrospective cohort study from 1 January 2005 to 30 June 2015 pertaining to outcomes of ESBL-producing *E. coli* and *K. pneumoniae* was conducted at the Chiang Mai University Hospital in Chiang Mai University. This study was approved by the ethics committee on human research of the Faculty of Medicine, Chiang Mai University of a waiver of informed consent for retrospective data collection under the condition of anonymously stored data collected. All methods were performed in accordance with the relevant guidelines and regulations. Patients were included if they were equal or greater than 18 years old and had a microbiologically documented infection with ESBL-producing *E. coli* or *K. pneumoniae* infection*.* Infection was defined according to the Center for Disease Control and Prevention (CDC) criteria^27^. Patients were excluded if they had received < 2 doses of the studied drug, i.e. LD colistin and carbapenems, received any other agents with activity against the offending ESBL isolate (e.g. aminoglycosides, fluoroquinolones, trimethoprim/sulfamethoxazole and fosfomycin), had other types of Gram-negative infections, received hemodialysis or renal replacement therapy. The recruited patients with ESBL infection were divided into two groups of LD colistin and carbapenems.

The carbapenems case was defined as a patient who had received any carbapenems, e.g. ertapenem, imipenem and meropenem in order to treat the documented ESBL infection for longer than 48 h and received only one course the treatment. Likewise, LD colistin case was defined as a patient who had been treated the documented ESBL infection with 300 mg of colistin base activity (CBA) once at the start of treatment course for longer than 48 h and treated only one course of colistin. The definitive therapy included patients receiving definitive monotherapy with LD colistin or carbapenems (imipenem/cilastatin, meropenem, ertapenem) for treatment of the infection with the ESBL-producing organism.

Dosage regimens of antibiotic were usually based on the respective hospital guidelines: LD colistin 300 mg of CBA once at the start of treatment course, and then 150 mg of CBA every 12 h, meropenem 1 g every 8 h, imipenem 500 mg every 6 h and ertapenem 1 g every 24 h. All doses were adjusted for renal function accordingly.

### Data collection

Patient data were collected through computerized medical records and patient chart review. The following data were obtained from medical records: age, gender, intensive care unit (ICU) admission during infection, Charlson score, underlying disease, source of infection (as documented in the medical record by the treating physicians), duration of positive cultures and results of antimicrobial susceptibility testing, length of stay, invasive mechanical ventilation, timing of antibiotic therapy, mortality status and nephrotoxicity.

### Outcome assessment

Three primary outcomes in this study were mortality rate at 30 days, clinical and bacteriological responses after the start of treatment. Thirty-day mortality was defined as death within 30 days of an ESBL-producing *E. coli* or *K. pneumoniae* infection. Clinical response of treatment was assessed by resolution or partial resolution of fever, leukocytosis, and local signs and symptoms of ESBL infections at the end of treatment. Clinical failure was defined as failure to meet all criteria for clinical response. Microbiological response was defined as obtaining two consecutive negative ESBL cultures from the site of infection after the initial positive culture, whereas microbiological failure was defined as persistence of the original causative organism in the subsequent specimen cultures. Nephrotoxicity was counted if patients developed any grades of renal failure based on RIFLE criteria.

### Statistical analysis

All statistical analyses were carried out using Stata software, version 14 (Stata-Corp, College Station, TX). Descriptive statistics were used to describe the data: frequencies and percentages for categorical variables and means with standard deviations for continuous variables. To compare two groups, Fisher’s exact test was used for categorical variables and independent t-test was used for continuous variables. A two-tailed test result with *P* value of < 0.05 was considered to be statistically significant. The rate of time to 30-day mortality, clinical failure and microbiological failure were evaluated using Cox regression model that controlled different variables between the two groups. Hazard ratios (HRs) were reported, with 95% confidence intervals (CIs). Potential confounders and interactions were added using a backward method. Additional adjustment for any variables with a *P* value < 0.20 on univariable analysis were included in an adjusted Cox regression model. In addition, all variables that demonstrated a trend toward association with outcomes were forced into the model at the discretion of the investigator and statistical significance was set at *P* ≤ 0.05. The proportional hazard assumption was checked for all models. Mortality rates of patients treated with LD colistin or carbapenems were compared using Kaplan–Meier curve and log-rank test. All tests were 2-tailed and *P* values ≤ 0.05 were used for statistical significance testing.

### Antimicrobial susceptibility testing

*E. coli* and *K. pneumoniae* were identified at a division of Clinical Microbiology, CMUH, using conventional cultures and biochemical methods. Antibiotic susceptibility was performed by disk diffusion method and minimal inhibitory concentrations (MICs) using E-test, encompassing nearly all important antibiotics^28^. The presence of ESBL was detected using the double-disc synergy test between clavulanate and third-generation cephalosporins (i.e. cefotaxime, cefpodoxime, ceftriaxone and ceftazidime). Antibiotic susceptibility and MICs were analyzed according to the CLSI^28^. Colistin susceptibility was determined by Vitek-2 automated method (bioMérieux, Marcy-L'Etoile, France)^23–26^. This study used colistin susceptibility (≤ 2 µg/mL) following the European Committee on Antimicrobial Susceptibility Testing (EUCAST) breakpoints^29^.

### Ethics approval and consent to participate

This retrospective cohort study was approved by the ethics committee on human research of the Faculty of Medicine, Chiang Mai University. (NONE-2558-02791).

## Data Availability

The datasets used and analyzed during the current study are available from the corresponding author on reasonable request.
